# Dr. Vasant Ramji Khanolkar: A Revolutionary Pathologist in India

**DOI:** 10.7759/cureus.68962

**Published:** 2024-09-08

**Authors:** Manoj Patil, Unnati Shirbhate

**Affiliations:** 1 Public Health, Datta Meghe Institute of Higher Education and Research, Wardha, IND; 2 Department of Periodontics, Sharad Pawar Dental College, Datta Meghe Institute of Higher Education and Research, Wardha, IND

**Keywords:** cancer, epidemiology, historical vignette, india, leprosy, medical education

## Abstract

Dr. Vasant Ramji Khanolkar is known as the Father of Pathology in India. His outstanding contributions include research on the epidemiology of cancer in India, leprosy, reproduction anatomy, and family planning practices in India. He had the lion's share in medical education in India and started the subjects of Biophysics and Applied Biology at Bombay University. He recognized the importance of basic science, experimental biology, biochemistry, biophysics, and numerology in medical education. He suggested many changes in the medical education curriculum of India and strengthened the research facilities in medical education. Dr. Vasant Ramji Khanolkar was honored with Padma Bhushan, a prestigious award by the Government of India.

## Introduction and background

Biography

Dr. Vasant Ramji Khanolkar was born on April 13, 1895, in a small village in the Konkan region of Maharashtra, India [1]. His father Ramji Dhondo Khanolkar was a renowned surgeon. Vasant Khanolkar probably got his interest in medicine and languages ​​from his father and decided to become a doctor at an early age. At the age of 17, he joined the Grant Medical College, Mumbai. He left for England in the same year and graduated from the University of London with a Bachelor of Science degree and a medical degree in 1918. In 1923, he received his master's degree (M.D.) in Pathology. During this time, he gained invaluable experience in basic science and medical research. He was the youngest Graham Research Scholar. Dr. Khanolkar was proficient in many languages. He could speak and read six Indian and four European languages [1,2].

## Review

Contributions to medical education in India

Returning to India, he started teaching pathology at the Grant Medical College to graduate and post-graduate students. He started a museum of various specimens useful for education. He started the systematic education of diagnostics. He also laid special emphasis on research in pathology. He suggested many changes in the medical curriculum so that Indian education does not fall short compared to the medical education in Western countries. He recognized the importance of basic science, experimental biology, biochemistry, biophysics, and numerology in medical education. He tried to spread this philosophy among his students. Dr. Khanolkar, as a teacher of diagnosis, gave a new direction to the study of this subject. Seth G. S. Medical College and K.E. M. Medical College have the honor of being an excellent medical institute at national and international levels. He started the subjects of Biophysics, Applied Biology, etc., in Bombay University [1-4].

Contributions to cancer research in India

During his teaching career, Dr. Khanolkar developed a special curiosity and interest in the study of cancer and leprosy. After retirement from the teaching profession, he joined the Tata Memorial Hospital as Chief Pathologist in 1941. He did research in cancer, leprosy, the anatomy of reproduction, and human genetics [2-5]. He was a pioneer of cancer research in India. During 1941-1951, he was engaged in tumor diagnosis at the Tata Memorial Hospital. His research topic was cancer in India. He studied hospital statistics to find out if there is cancer in India and what are the major types. He studied some factors/habits and epidemiology of cancer. He explored the relationship between cancer incidence and cultural practices. He explored the correlations between certain habits and customs among Indians and cancers in specific parts of the body. To investigate these connections, extensive surveys were conducted across various regions of the country. He was among the first to suggest that chewing tobacco and betel nut might cause oral cancer.

Between 1941 and 1951, Dr. Khanolkar concentrated on various aspects of cancer, including biochemical, cytological, genetic, histological, and statistical studies. His research revealed that the incidence of cancer in India was much higher compared to Western countries. He considered statistical analysis of cancer morbidity, mortality, and the occurrence of different cancer types crucial for understanding and treating the disease. He argued that while not all cures were known, effective treatment protocols could lead to cures in many cases. He also emphasized the importance of maintaining thorough records and follow-up systems to advance cancer research and treatment knowledge [5]. His research papers in this context were published in many national and international journals [2,5-7]. As a credit for his pioneering work on cancer in India, Dr. Khanolkar was awarded the International Union Against Cancer membership in 1947. Under his leadership, the Indian Cancer Research Center (ICRC) was started in 1952 which received financial support from the Rockefeller Foundation, the World Health Organization, and many other global organizations [1].

Contributions to other medical research in India

Apart from cancer, Dr. Khanolkar also researched leprosy. He was involved in working on the anatomy of reproduction. It was through his initiative that the Indian Council of Medical Research appointed a committee to conduct a scientific study of family planning. He was the chairman of this committee for seven years. He founded the Indian Association of Pathologists and Microbiologists. He has published three books and over a hundred research papers on cancer and leprosy [1,2,8].

Initiatives for starting the institutes of repute

Dr. Khanolkar retired as Director of the Indian Cancer Research Centre (ICRC) in 1963. He had a role in planning the All India Institute of Medical Sciences, New Delhi; the Bhabha Atomic Research Center; the Post-Graduate Institute for Medical Research in Chandigarh and Institute for Research in Reproduction [5]. 

Honors

The government of India honored Dr. Khanolkar with Padma Bhushan in 1955. He was the founder and president of the Indian Association of Pathology and Microbiology, chairman of the International Cancer Research Commission, director of the Indian Cancer Research Center, member of the World Health Organization Committee on Cancer and Leprosy and Vice-Chancellor of the University of Mumbai. The National Academy of Sciences started an elocution competition in his memory [2,8].

Death

On October 29, 1978, Dr. Khanolkar died at King Edward Memorial Hospital Mumbai [7]. 

List of scientific publications reflected in PubMed

Around 50 articles authored or co-authored by Dr. V. R. Khanolkar are still reflected in PubMed (Table 1).

**Table 1:** List of scientific publications authored by Dr. V. R. Khanolkar

PMID	Title	Authors	Citation	First Author	Journal/Book	Publication Year
19970956	Granular Cell Myoblastoma	Khanolkar VR. [9]	Am J Pathol. 1947 Sep;23(5):721-39.	Khanolkar VR	Am J Pathol	1947
21026999	Bowen's disease of the conjunctiva	Khanolkar VR. [10]	Am J Ophthalmol. 1946 May;29:515-9. doi: 10.1016/0002-9394(46)91507-3.	Khanolkar VR	Am J Ophthalmol	1946
20982093	Genetic theories of Rh blood types	Khanolkar VR, Sanghvi LD. [11]	Ann Eugen. 1946 Apr;13:7-14. doi: 10.1111/j.1469-1809.1946.tb02337.x.	Khanolkar VR	Ann Eugen	1946
13255883	Gamma-Sitosteryl glycoside in tobacco	Khanolkar VR, Panse TB, Divekar VD. [12]	Science. 1955 Sep;122(3168):515-6. doi: 10.1126/science.122.3168.515-a.	Khanolkar VR	Science	1955
20989960	Melanoma of the alveolus	Gharpure VV, Baliga AV, Khanolkar VR. [13]	Indian Physician. 1945 Jan;4:18.	GHARPURE VV	Indian Physician	1945
20989961	Liposarcoma of bone	Khanolkar VR. [14]	Indian Physician. 1945 Jan;4:19.	Khanolkar VR	Indian Physician	1945
13755627	Diagnosis of leprosy	Khanolkar VR. [15]	Triangle. 1960 Oct;4:251-9.	Khanolkar VR	Triangle	1960
13513145	Dimorphous polyneuritic leprosy	Cochrane RG, Khanolkar VR. [16]	Indian J Med Sci. 1958 Jan;12(1):1-9.	COCHRANE RG	Indian J Med Sci	1958
14087516	Studies on hemoglobin variants and glucose-6-phosphate dehydrogenase in Indian sheep and goats	Khanolkar VR, Naik SN, Baxi AJ, Bhatia HM. [17]	Experientia. 1963 Sep;19:472. doi: 10.1007/BF02150653.	Khanolkar VR	Experientia	1963
14128910	Recent advances in our knowledge of mycobacterium leprae	Khanolkar VR. [18]	Indian J Med Res. 1964 Feb;52:139-50.	Khanolkar VR	Indian J Med Res	1964
18907568	The effect of foster-nursing on the morphology of the mammary glands in mice	Khanolkar VR, Ranadive KJ. [19]	J Pathol Bacteriol. 1947 Oct;59(4):593-603. doi: 10.1002/path.1700590410.	Khanolkar VR	J Pathol Bacteriol	1947
20989968	Carcinoma of the base of the tongue	Mody KP, Athle, Khanolkar VR. [20]	Indian Physician. 1945 Mar;4:58.	Mody KP	Indian Physician	1945
21025577	The blood-bank at the Tata Memorial Hospital, Bombay	Khanolkar VR. [21]	Ind Med Gaz. 1946 Jan;81(1):32-9.	Khanolkar VR	Ind Med Gaz	1946
13267409	Habits and customs as causal factors of cancer	Khanolkar VR. [22]	Schweiz Z Pathol Bakteriol. 1955;18(4):423-8. doi: 10.1159/000160797.	Khanolkar VR	Schweiz Z Pathol Bakteriol	1955
14363805	Smoking and chewing of tobacco in relation to cancer of the upper alimentary tract	Sanghvi LD, Rao KC, Khanolkar VR. [23]	Br Med J. 1955 May 7;1(4922):1111-4. doi: 10.1136/bmj.1.4922.1111.	Sanghvi LD	Br Med J	1955
15403127	Data relating to seven genetical characters in six endogamous groups in Bombay	Sanghvi LD, Khanolkar VR. [24]	Ann Eugen. 1949 Oct;15(1):52-76. doi: 10.1111/j.1469-1809.1949.tb02422.x.	Sanghvi LD	Ann Eugen	1949
20294739	Some facts about cancer	Khanolkar VR. [25]	J Indian Med Assoc. 1946 Nov;16(2):37-43.	Khanolkar VR	J Indian Med Assoc	1946
20991334	Science and the student	Khanolkar VR. [26]	Indian Physician. 1945 Apr;4:80-9.	Khanolkar VR	Indian Physician	1945
14050624	Experimental studies on the etiology of cancer types specific to India (a) oral cancer (B) Kangri cancer	Ranadive KJ, Gothoskar SV, Khanolkar VR. [27]	Acta Unio Int Contra Cancrum. 1963;19:634-9.	Ranadive KJ	Acta Unio Int Contra Cancrum	1963
20984880	A doctor and his books	Khanolkar VR. [28]	Indian Physician. 1946 May;5:101-9.	Khanolkar VR	Indian Physician	1946
13853372	Care of the aged in India	Khanolkar VR. [29]	Excerpta Med. 1959 Dec;[XX] 2:523-6.	Khanolkar VR	Excerpta Med	1959
21017325	The susceptibility of Indians to cancer	Khanolkar VR. [30]	Indian J Med Res. 1945 Oct;33:299-314.	Khanolkar VR	Indian J Med Res	1945
13649428	Oral cancer in India	Khanolkar VR. [31]	Acta Unio Int Contra Cancrum. 1959;15(1):67-77.	Khanolkar VR	Acta Unio Int Contra Cancrum	1959
14455560	Breast cancer in Bombay	Khanolkar VR. [32]	Acta Unio Int Contra Cancrum. 1961;17:903-4.	Khanolkar VR	Acta Unio Int Contra Cancrum	1961
14408805	A quest after cancer cures	Khanolkar VR. [33]	Acta Unio Int Contra Cancrum. 1959;15(Suppl 1):151-3.	Khanolkar VR	Acta Unio Int Contra Cancrum	1959
21064881	Leiomyosarcoma in the tonsillar area	Jussawalla DJ, Gharpure VV, Khanolkar VR. [34]	Indian Physician. 1945 Mar;4:60.	Jussawalla DJ	Indian Physician	1945
18118927	Reticulum cell sarcoma of bone	Khanolkar VR. [35]	Arch Pathol (Chic). 1948 Nov;46(5):467-76.	Khanolkar VR	Arch Pathol (Chic)	1948
20989970	Dendritic adenocarcinoma of sub-maxillary gland	Khanolkar VR, Meher-Homji DR. [36]	Indian Physician. 1945 Mar;4:61.	Khanolkar VR	Indian Physician	1945
13219428	[Geography and cancer in India]	Khanolkar VR. [37]	Bull Assoc Fr Etud Cancer. 1954;41(2):247-63.	Khanolkar VR	Bull Assoc Fr Etud Cancer	1954
14447211	Submucous fibrosis of the palate in diet-preconditioned Wistar rats. Induction by local painting of capsaicin--an optical and electron microscopic study	Sirsat SM, Khanolkar VR. [38]	Arch Pathol. 1960 Aug;70:171-9.	Sirsat SM	Arch Pathol	1960
20987978	Prognosis in cancer of the female breast	Khanolkar VR. [39]	Indian J Surg. 1945 Mar;7:30-9.	Khanolkar VR	Indian J Surg	1945
13913462	Submucous fibrosis of the palate and pillars of the fauces	Sirsat SM, Khanolkar VR. [40]	Indian J Med Sci. 1962 Mar;16:189-97.	SIirsat SM	Indian J Med Sci	1962
13598476	In vitro studies on human leprosy	Ranadive KJ, Nerurkar RV, Khanolkar VR. [41]	Indian J Med Sci. 1958 Oct;12(10):791-6.	Ranadive KJ	Indian J Med Sci	1958
13755628	Functional components of chemically induced mammary cancer	Khanolkar VR. [42]	Acta Unio Int Contra Cancrum. 1961;17:45-54.	Khanolkar VR	Acta Unio Int Contra Cancrum	1961
13990731	Studies of pathogenicity of the ICRC bacillus isolated from human lepromatous leprosy	Ranadive KJ, Bapat CV, Khanolkar VR. [43]	Int J Lepr. 1962 Oct-Dec;30:442-56.	Ranadive KJ	Int J Lepr	1962
13896638	Cytological study of three cell lines derived from a human lipogenic sarcoma	Gangal SG, Ranadive KJ, Khanolkar VR. [44]	Indian J Med Res. 1961 Sep;49:755-67.	Gangal SG	Indian J Med Res	1961
14032286	Pathogenesis of certain anomalies of sex in man	Khanolkar VR, Shah PN, Mahajan DK. [45]	Indian J Med Sci. 1962 Dec;16:995-1005.	Khanolkar VR	Indian J Med Sci	1962
13462567	Studies on B vitamins and estrogens in oral cancer	Sathe VR, Talageri VR, Khanolkar VR, Panse TB. [46]	Indian J Med Res. 1957 Jul;45(3):401-9.	Sathe VR	Indian J Med Res	1957
14203318	Immunologic studies with mycobacterium leprae and an acid-fast mycobacterium cultivated from human leprosy nodules	Rao SS, NadkarniJS, Khanolkar VR. [47]	Int J Lepr. 1964 Apr-Jun;32:103-16.	Rao SS	Int J Lepr	1964
14914589	Cancer in India in relation to race, nutrition and customs	Khanolkar VR. [48]	Acta Unio Int Contra Cancrum. 1951;7(1 Spec. No.):51-60.	Khanolkar VR	Acta Unio Int Contra Cancrum	1951
14824924	Cancer in India in relation to race, nutrition, and customs	Khanolkar VR. [49]	J Natl Cancer Inst. 1950 Dec;11(3):640-2.	Khanolkar VR	J Natl Cancer Inst	1950
14447210	Histological, electron-microscope and enzyme studies of submucous fibrosis of the palate	Sirsat SM, Khanolkar VR. [50]	J Pathol Bacteriol. 1960 Jan;79:53-8. doi: 10.1002/path.1700790107.	Sirsat SM	J Pathol Bacteriol	1960
20992836	Diagnosis of bone tumours by aspiration; a review of 80 cases	Khanolkar VR, Nerurkar RV. [51]	Indian Physician. 1946 Jun;5:125-35.	Khanolkar VR	Indian Physician	1946
13864709	Growth characteristics of an acid-fast Mycobacterium isolated from human lepromatous leprosy	Bapat CV, Ranadive KJ, Khanolkar VR. [52]	Int J Lepr. 1961 Jul-Sep;29:329-42.	Bapat CV	Int J Lepr	1961
13366411	Use of fluorescence microscopy in the diagnosis of leprosy	Khanolkar VR, Nerurkar RV. [53]	Indian J Med Res. 1956 Jul;44(3):397-402.	Khanolkar VR	Indian J Med Res	1956
21012451	Cancer in relation to usages; three new types in India	Khanolkar VR, Suryabai B. [6]	Arch Pathol (Chic). 1945 Nov-Dec;40:351-61.	Khanolkar VR	Arch Pathol (Chic)	1945
13415683	Normal levels of urinary excretion of estrogens, pregnanediol and 17-ketosteroids in adult women and estrogens and 17-ketosteroids in men	Patwardhan VV, Panse TB, Khanolkar VR. [54]	Indian J Med Sci. 1957 Jan;11(1):4-10.	Patwardhan VV	Indian J Med Sci	1957
13913463	The effect of arecoline on the palatal and buccal mucosa of the Wistar rat. An optical and electron microscope study	Sirsat SM, Khanolkar VR. [55]	Indian J Med Sci. 1962 Mar;16:198-202.	Sirsat SM	Indian J Med Sci	1962
14447212	Submucous fibrosis of the palate. Induction by local painting of capsaicin in Wistar rats treated with desoxycorticosterone acetate--an optical and electron microscopic study	Sirsat SM, Khanolkar VR. [56]	Arch Pathol. 1960 Aug;70:180-7.	Sirsat SM	Arch Pathol	1960

## Conclusions

Dr. Vasant Ramji Khanolkar played a key role in medical education and research in India. His contribution to the field of pathology is remarkable. Even then, very limited literature is available online related to his work and research.
